# Supplementary material for: Providing Housing First services for an underserved population during the early wave of the COVID-19 pandemic: A qualitative study
Source: PLoS One. 2022 Dec 1;17(12):e0278459. doi: 10.1371/journal.pone.0278459 (PMC9714853; doi:10.1371/journal.pone.0278459)
Supplement: S1 File — (DOCX) [file pone.0278459.s001.docx]

**Supplementary Table 1. Individual Interview Guide: TORONTO AH/CS-QUALI’s COVID-19 sub-study with Front-line Housing First Providers.**

**Part II. COVID-19-related questions^1^ concerning challenges and adjustments HF providers experienced in providing support services to HF users, and the impact of the epidemic on HF providers’ well-being.**

**Challenges and adjustments experienced in providing support services to Housing First users**

1. What kind of challenges are you experiencing regarding the provision of services to your clients now during the COVID-19 epidemic?
2. What kind of adjustments have you implemented to support your clients in time of this COVID-19 epidemic?
3. What would you say are the most important priority or needs for your client during the COVID-19 pandemic period? And How are you supporting them?

**Needs of the Housing First services users and support given**

1. Do you know if your clients are receiving any additional social, health, or economic support within and outside of the HF program?
2. Access to food
3. Access to health (More Mental health services?)
4. Self-distancing
5. Access to sanitation
6. Access to income supports (e.g. – Canada Emergency Response Benefits)
7. Access to reliable health and prevention information on COVID-19
8. Access to the internet
9. What is the housing quality of your clients? For example, Air Conditioning, heating, a functioning kitchen, and washroom, etc.

- How are you supporting it?

1. Have any of your At Home clients received cell phones/tablets provided by any Government or private services?

- If so, who has provided them and who is paying for the monthly access fees?

**Support that Housing First providers receive during the covid-19 pandemic, lessons learned, and recommendations**

1. Is your agency receiving any economic relief from the Government to support COVID-19 issues/funding for your At Home clients?
2. What would you say have been the main learning or lessons from these Epidemic constraints (for your clients, your agency, and yourself as case manager provider?
3. What would you ask the local government to provide or do for homeless people outside your clients?

**Impacts of the covid-19 pandemic on the HF providers' well-being**

1. How this epidemic is affecting you as a case manager provider and person?
2. And are you receiving any support to help cope with any distress or other issue?

We are at the end of the interview but if there is anything else you would like to add that was not covered could you share that now?

**End of the interview**

This is the end of your interview. Thanks again for participating and sharing your experience and knowledge with us

Please, let me know if you have any questions related to this interview.

________________________________

1. These questions were initially used to guide the semi-structured conversations with study participants. They were based on our research objectives. Given the iterative nature of the qualitative inquiry, these questions evolved over the interview process.
