# Supplementary material for: Providing Housing First services for an underserved population during the early wave of the COVID-19 pandemic: A qualitative study
Source: PLoS One. 2022 Dec 1;17(12):e0278459. doi: 10.1371/journal.pone.0278459 (PMC9714853; doi:10.1371/journal.pone.0278459)
Supplement: Attachment — Submitted filename: Response to Reviewers.docx [file pone.0278459.s003.docx]

**Response to Reviewers’ Comments**

We thank the reviewers for their helpful feedback, comments and suggestions, which helped to improve the quality, readability and impact of the manuscript.

| **Reviewers’ Comments** | **Response** |
| --- | --- |
| **Reviewer #1:** |  |
| This qualitative study examined the experiences of service providers working in Housing First (HF) settings in Toronto, Ontario, Canada. This analysis is a sub-study from a rigorous, ongoing parent study of the long-term impacts of HF using both quantitative and qualitative methods. The manuscript offers insights into what it is like to work in HF programs during the pandemic, including the challenges strategies implemented to support service users, impacts on providers and lessons learned. Its novelty lies in its focus on the HF service provider experience for which, at this point in the pandemic, there are no known studies of HF providers – a perspective that was also somewhat under-examined prior to the pandemic. Further, this is an essential workforce that has been working throughout the pandemic (despite fears of becoming unemployed, according to this study), often in the shadows on the more traditional healthcare system. Accordingly, this study offers some validation of this sector’s hard work, including the challenges of the job. The manuscript has potential to be an important source of information on what this workforce experienced early on in the pandemic in Canada and offer valuable insights for the recovery to come, as well as future public health emergencies. | Thank you for your encouraging and positive feedback on this paper’s value and potential to further strengthen the response role of HF services and frontline workers during public health emergencies and beyond. |
| However, there are several issues that would first need to be addressed. First, the major issue here is the length of the results section, which is particularly cumbersome. Its current structure also leaves more to be desired in terms of a synthesis that would allow readers to efficiently extract key findings (described further below). | Thank you for this comment, which made us more reflective and critical of the presentation and synthesis of findings. We have revised the results and synthesised them in a more meaningful way for the audience (p 15-37). |
| As such, the results need to be condensed substantially (less is more here) and the overlap in a number of themes presents a feasible path to doing this. | We have revised the themes, grouping together the more salient and informative ones, and being mindful to avoid overlap and repetition. |
| Second, much of the discussion involves a summarization of the findings. It would be more useful to focus on how the findings fit with other related research (suggestions made below), highlight the implications of key findings, speak to future avenues for research, and devote more space to discussing the policy recommendations. | Thank you for these comments. We revised the discussion by highlighting the key findings and how they align or differ with results observed in existing studies, as at this point, additional evidence has been published on the pandemic's impacts and the role of support services in supporting socio-economically excluded populations (p 37-46). Also, we have strengthened the content on the implication of our findings regarding future research, policy and practice (Table 1, p 47-49). |
| Third, the study design requires more of a rationale as to why it was selected (not the study’s qualitative methods, but its recruitment and sample size). These comments are explained in more detail below, along with a number of minor points. | Thank you for this observation. We have provided detailed information on the rationale related to participants’ selection and sample size in the “Methods section”(p 9-11) In summary, the three main HF community-based organizations were selected as they were the first organization that provided HF services for people experiencing chronic homelessness and mental disorders enrolled in the At Home/Chez Soi (AH/CS) randomised controlled trial at the Toronto site. Among the three organizations, one provides Assertive Community Treatment (ACT) for people experiencing homelessness and high-need level for mental health services, a second one offers Intensive Case Management (ICM) for people experiencing homelessness with a moderate- need level for mental health services, and the third provides ICM with an ethno-racial focus for people experiencing homelessness with a moderate-need level for mental health services interested in receiving racially and cultural-appropriate care. We employed a convenience and purposive sampling strategy to recruit three Case Managers employed at each of the three HF organizations as part of the overarching study, Toronto At Home/ Chez Soi: Qualitative follow-up study (TORONTO AH/CS-QUALI STUDY), which aimed to identify factors, life experiences, structural challenges and unmet needs that act as barriers for HF clients to achieve more sustainable social, health and community integration improvements over longer-term horizons.  The decision to include Case Managers rested on the fact that they work collaboratively, closely and frequently with HF clients to assess their housing, health, social and economic needs and facilitate, coordinate, and advocate for access to appropriate services and resources to meet such needs on an ongoing basis and over time. Therefore, they were and are best placed to evaluate facilitating and constraining factors faced by the HF program to appropriately meet the needs of HF clients during and beyond public health emergencies. Furthermore, they can provide valuable insights into HF clients' conditions, status, experiences, challenges and opportunities due to their close alliance and long-term working relationships. The decision regarding the number of recruited participants (three case managers for each HF organization, for a total of nine participants) rests on the fact that this represented a sufficient number to gain comprehensive within-agency insights on the topics studied. At the same time, the overall sample of nine was sufficient for reaching discourse saturations. Finally, we were very lucky to successfully recruit nine Case Managers during the first wave of the COVID-19 pandemic, when service disruptions were at their higher level, particularly in social and health service settings where frontline staffing was already stretched. |
| Abstract:  - The conclusion needs more of a ‘so what?’ element | Thank you for this comment. In the revised abstract, we have now included the so ‘so what?’ statement (Abstract section, p 3-4) |
| Introduction  - Not familiar with the term “Equity-seeking groups,” can guess its meaning, but please define (further, particularly important to make clear who you’re referring to given the next sentence with notes serious consequences) | - Thank you for the observation. We acknowledge that the term “Equity-seeking groups” could be confusing and interpreted by each reader differently. Yet, it refers to the umbrella of communities that face significant collective challenges in participating in society because of barriers to equal access, opportunities and resources due to disadvantage and discrimination, and actively seek social justice, such as those with experiences of chronic homelessness and other intersecting factors, such as discrimination, stigma, mental illness. <https://canadacouncil.ca/glossary/equity-seeking-groups>). To avoid confusion, we have replaced it with the specific population we are referring to (p 6). |
| - The case for the value of the research can be strengthened in the final paragraph of the introduction; objective 4 gets at this a little bit, but it needs to be clearer what understanding “the challenges, experiences, and lessons learned” during wave 1 of the pandemic will do for the HF sector/research community. The first two sentences on why qual. methods are valuable can also probably be scrapped, or moved to methods at the very least. | Thank you for these important comments. We have clarified the value of the present study, for HF clients, providers, and communities, social and health sectors (p 7-8).  We have moved the qualitative methods statement to the “Methods section” (p 9). |
| Methods  - It’s a small sample drawn from 3 organizations. This is not necessarily a weakness of the study, though a rationale for why data collection was set or stopped at 9 participants is certainly needed. Providing a justification of 3 organizations is probably warranted as well, as opposed to recruiting 9 participants from a single organization, for example. | Thank you for this comment. As noted above, we have provided further detail on the rationale for the sample selection and size in the “Methods section” (p 9-11). |
| - Very minor: make clear that the sub-study received REB approval and that it is not just the parent TORONTO AH/CS-QUALI STUDY | We have made clear that while our study is linked to the original At Home/Chez Soi (AH/CS) randomised controlled trial at the Toronto site (implemented in 2009), the TORONTO AH/CS-QUALI STUDY is an independent study which received independent ethics approval in December 2019 from the Research Ethics Board (REB) at Unity Health Toronto. Furthermore, as the implementation of the TORONTO AH/CS-QUALI STUDY coincided with the first wave of the COVID-19 pandemic, we received authorization from the REB to conduct this COVID-19 related sub-study. Therefore, we have made it clear in the “Methods section” (p 11). |
| Kudos for providing a good value honorarium to HF workers (it’s not common enough practice when conducting research on providers and this was a workforce, which was dealing with a barrage of pandemic-related issues and feeling undervalued when the study was conducted, so this was a considerate decision). No action needed on this comment. | Thank you for this positive observation. Despite having a limited budget, we recognized the dare circumstances that frontline workers were facing during the first months of the pandemic to support their clients while at the same time exposing them to the risk of contracting COVID-19 and even remaining unemployed. Therefore, we found it important to recognise their time spent participating in our study with a small honorarium. |
| - “To further enhance the privacy and security of the data collected, we recorded interviews using an external audio-recording device rather than using the internal audio- record features of the Zoom platform.” Poor transcriber(s). No action needed on this comment. | Thank you. We agree with your comment. |
| - Can probably remove the word “solid,” which is a little on the casual side (though I have no doubt the scientist did have a solid research background in the noted areas). | Thank you for this observation. We have removed it accordingly. |
| Describe what was the “initial tree-styled AF” - Define first-, second-, and third-grade themes, and their hierarchy (if applicable) | Thank you for the observation. We expanded the details of the theme’s framework in the “Methods section” (p 13-14). |
| **Results** |  |
| Is there any more data available on the characteristics of the sample? For example, gender, ethnicity, age, education, et cetera. Also, provide range for participants’ HF work histories: “All HF frontline workers had worked with HF clients for more than three years.” | Thank you for this observation. We expanded the description of the participants by only providing information on their self-identified gender and the amount of time employed with HF organizations (p 15). We cannot provide detail on self-identified ethno-racial status and age as it will increase the risk of identification of participants from such a small sample. The participating HF agencies are well recognized by HF clients, other support organizations, and internal and external stakeholders. Providing such details could increase the identification risk, which may lead to a breach in confidentiality and anonymity assured to participants and governed by the REB. |
| - Opening quote of theme 1: “The reality is, is it is, everything is very limited…” Might the participant have said “as it is”? | Thank you for the suggestion. We corrected potential misspellings in the transcribed interviews. |
| The results and their thematic structure are LONG (by my count: the results are currently 24.5 pages, excluding figure 1). Their length presents two key problems: [1] There is overlap and redundancies throughout the results section and [2] It’s hard to tell what is most important and how the findings between first-grade themes intersect. As an example of the former, sections 1.1 and 1.2 both discuss the challenges caused by phone communication (e.g., “…many clients were reluctant to disclose their mental health needs by phone…” in section 1.1 and “HF frontline workers shared that many of their clients were reticent or did not want to speak about personal matters or health issues via phone or virtually” in section 1.2). As an example of determining what is most important, the third-grade themes in the second main theme describe 21 different strategies used by providers. However, commentary on what worked well, what did not, what it was like for providers to implement the strategies, what was perceived as most important are not often commented upon.  Further, what might be innovative (e.g., requesting a medical prescription for air conditioning – very interesting, would like to know more about how providers found this, extent to which they felt this was effective) gets lost in the mundane (e.g., this foundational component of case management: “They were also constantly advocating with key liaison actors (e.g., social workers, nurses) in primary care and hospital-based care services to ensure the access and delivery of appropriate care, including hospital admissions and continuity of care for non-COVID-19 related issues.”). These issues make me wonder if the analytic approach had a sufficient synthesis built into it, as there remains a lot of data presented descriptively. | We agree with the comment that the results were too long and required further synthesis and readable structure. We revised the data and results accordingly, making them more analytical than descriptive. The results are presented in a more meaningful and concise manner. We also included participants’ spoken words within the results’ text rather than present them in tables (p 15-37).  We have given special attention to what worked well, what didn't, and the experiences of providers implementing such response strategies, while highlighting innovative findings such as advocating for medical prescriptions for air conditioning (p-26). |
| - Minor: Revise for clarity, as alcohol consumption is a type of substance use (“in response to the increased substance use, and alcohol consumption”) | Thank you for this observation which we agree with. We amended the sentence accordingly. |
| - Typo: 3.3 coping strategies | We proofed the manuscript and corrected all misspelling or typo errors. |
| **Discussion** |  |
| - The discussion is mostly focused on summarizing the findings, with a sprinkling of contextualization using recent research. However, there are some omissions that could be considered by the authors (prioritizing other qualitative studies in the articles suggested below):  Kaur et al. (2021). Provision of services to persons experiencing homelessness during the COVID-19 pandemic: A qualitative study on the perspectives of homelessness service providers. Health Social Care in the Community  Levesque et al. (2021). Understanding the Needs of Workers in the Homelessness Support Sector. Homeless Hub report.  Peters et al. (2021). A systematic review and meta-synthesis of qualitative studies that investigate the emotional experiences of staff working in homeless settings. Health Social Care in the community (not specific to pandemic, though some of your findings match up with this)  Pixley et al. (2021). The role of homelessness community based organizations during COVID?19. Health Social Care in the Community (not specific to HF, though some of your findings match up with this)  Parkes et al. (2021). ‘They already operated like it was a crisis, because it always has been a crisis’: A qualitative exploration of the responses of one homeless service in Scotland to the COVID-19 pandemic (not specific to HF, though some of your findings match up with this)  Another grey evidence source is CAEH’s COVID-19 information exchange (https://caeh.ca/homelessness-sector-covid-19-information-exchange/), which provides evolving data on the impacts of the pandemic on the homelessness/housing service sector in Canada. It would be interesting to see if what they were hearing from providers across the country during the first wave (again, more broader scope than only HF providers) is consistent with your sample. Food for thought. | We thank you for the helpful comments regarding the discussion, which have led to a more refocused and strengthened manuscript. We thank you for the suggested literature, which we have included alongside other recent references in the revised manuscript (p 37-46). |
| - The first limitation is NOT a limitation and needs to be removed. It is invalidating toward the experiences of HF workers and the authors have not suggested that the findings represent HF service users, so the point is irrelevant. | We agree with your comment and have removed the sentence. |
| Practice and Policy Recommendations - Clarify that “social housing programs” do not refer to “social housing” - The recommendations are fairly comprehensive and general. Three considerations on which I would encourage the authors to reflect: [1] Do the recommendations overstep the findings? [2] Is a qualitative study of 9 participants sufficiently positioned to make such sweeping recommendations? and [3] What meaningful actions can HF programs take to support clients and staff during the pandemic? (note: HF programs are not resourced and/or positioned to make some of the transformative changes that are recommended here; what are the small, but potentially significant changes that HF programs could implement) | Thank you for these helpful observations. We reviewed our recommendations (Table 1, p 47-49), paying particular attention to scaling them down to be more actionable for HF programs. Also, we highlighted in the recommendations, the important role that HF programs play in responding to public health emergencies with further support from local and national governments to strengthen their pandemic response and post-pandemic recovery capacity. |
| There are a number of typos in Figure 2 | Thank you. We replaced existing Figure 2 with a table (Table 1) and proofed it accordingly. |
| **Other** |  |
| - Be consistent with language for HF recipients through the paper (i.e., clients or service users) | Thank you for this observation. We revised the language to ensure consistency throughout the manuscript. |
| **Reviewer #2:** |  |
| - The manuscript “Providing Housing First services for an underserved population during the COVID-19 pandemic: A qualitative study”, examines the experiences of housing first providers during the first wave of the pandemic in Toronto, ON, Canada. This manuscript is well-written, the findings are interesting and have important implications for the provision and implementation of Housing First. | We thank you for this positive observation and reassuring comment. |
| There are a few minor adjustments I would recommend. The Results section should be refined more to highlight the most important themes. | We agreed with your comment regarding the need to synthesize results and present them more analytically. In response to this comment which was shared by another reviewer, we revisited the data and revised the findings, presenting the most salient and novel ones (Figure 1). |
| The implications of these central findings could be developed more in the Discussion. The Discussion should more clearly convey the authors' core conclusions. | Thank you for your suggestion. We revised the discussion, being mindful of highlighting the salient findings in relation to existing evidence (p 37-46). We also elaborated further on their implications for research, policy and practice (Table 1, p 47-49). |
| **Results** |  |
| In the Results section the reader is directed to Tables 1-4. These tables are somewhat difficult to read and interpret. The authors should consider either removing the tables and re-integrating excerpts in the text, and/or revising the tables so that they are less packed with text and easier to interpret. It should be easier for the reader to make the link between the excerpt and theme, and to understand the excerpts. | Thank you for this comment. We integrated examples of participants’ spoken words (excerpts) to illustrate the study’s main findings within the text rather than presenting them on a table. Therefore, the existing tables containing the quotes have been removed. |
| A little more signposting is needed throughout the Results. For example, on p. 11 I was confused to discover that “Challenges that HF frontline providers faced in serving HF program clients during the COVID-19 pandemic” was just the overarching theme, and that the authors would be instead presenting the sub-themes in detail. Make clear this is an introductory paragraph and not a substantive theme (and throughout the remaining sections too). | We revised the findings and presented the most salient themes in a more analytical, concise and structured way, focusing on the main or overarching themes (p15-37). |
| This leads me to another issue, if including an excerpt, it needs to be followed by an explanation, so either explain here and throughout the Results, or include with interpretation in another place in the text. For example, on p. 12 I’d like to hear more about the excerpt, why was virtual a problem for clients? On p. 27, a supporting excerpt in the section on “High psychological stress and anxiety” would go a long way. | Thank you for this observation. We included participants’ excerpts throughout the findings section to illustrate the salient themes (p15-37). . |
| I really like Figure 1. and wonder why it isn’t integrated better in the results or Discussion sections. The authors should consider this as a basis for developing their overarching framework. | Thank you for this feedback. We used the revised Figure 1 to illustrate the main identified themes and present the findings aligned with it, but such figure was removed. We also developed a Figure (Figure 1) to visually illustrates the salient findings and the underlying direct or indirect relationships. |
| Themes are plentiful, maybe some are more important or hold stronger implications for practice than others? I would suggest that the authors consider reducing or integrating themes to highlight the most important ones. For example, very important themes include the strategies implemented to support HF clients’ housing needs, and the implementation of housing-related adjustments. | We agree with your comment that the more salient, informative and novel findings should be reported. We revised the data and presented the main findings (p15-37) in a more analytical and concise way, highlighting the findings regarding the strategies implemented to support HF clients' housing needs. |
| Commentary on “implementation of housing-related adjustments” on p. 33 should be expanded particularly around reducing the reliance on drop-in shelter services for people in housing. This is a common issue in the field but not represented in the literature to date, as far as I know. | Thank you for this helpful feedback. We agree that this is an important finding that needs to be highlighted, as, unfortunately, it is a frequently reported issue that has not been appropriately addressed and understood. |
| Themes read as categories that map onto the research objectives rather than as themes representative of patterns in the data. Adjust theme names so they tell the reader how they inform the research objectives. | Thank you for this observation. We adjusted the themes based on the re-analysed and revised “Results section” and identified them more informatively. |
| **Minor comments, suggestions** |  |
| There are some interesting findings this reviewer would like to hear more about, on p. 22 how did frontline workers increase harm reduction education and support? And were their advocacy efforts successful? P. 25 more discussion on the provision of temporary accommodation in the form of homeless shelters/hotel programmes. P. 29, really interesting that frontline workers accessed support through employment support programmes, this is not available to HF workers in many other contexts/countries | Thank you for this feedback. We revised the data and provided additional insights on increasing harm reduction supports (p 22-23) and providing temporary accommodation for some HF clients that may be needed during a pandemic (p 25-27).  Furthermore, we agree that the findings that HF frontline workers received stress, psychological and emotional decompression support through their employment benefit programs should be highlighted as it may guide the implementation of similar supports to HF workers in other settings, not only during the pandemic but also beyond. HF workers help clients with complex and intersecting health, social and economic needs; therefore, it is vital to provide them with appropriate support to help them cope with the indirect or direct effects of their job on their psychosocial well-being . We highlighted in the recommendation (Table 1, p 47- 48). |
| **Discussion** |  |
| Reduce repetition of results, build a foundation in alignment with existing literature, and draw out conclusions, and implications. E.g. p. 41 the conclusion at the end of paragraph one about a UK study. Draw this out a little more to conclude based on the findings of this study together with existing research. The use of assertive language should help with making more concrete conclusions. | Thank you for this comment, which contributed to strengthening the discussion, recommendations and conclusions. We re-structured the discussion section, avoiding repetition and aligning our findings with existing evidence, thus providing more concrete recommendations and conclusions (p, 37-49). |
| With a little engagement with the HF literature, the authors should present their recommendations for HF implementation. For example, should HF programmes have an emergency/crisis readiness component? Were there failures identified that should be addressed? What kind of encompassing policies would help with HF crisis readiness? How do findings inform policies on harm reduction? | Thank you for the suggestion and reflective questions. We discusses substance use/harm reduction within the “Discussion” section and in the revised the recommendations, placing particular attention on the advised actions that could be implemented at the HF program level to further build further their role in responding to public and social emergencies and beyond (Table 1, p 47- 48). |
| Develop the policy recommendations a bit more by giving examples or comparing proposed changes with existing approaches. For example in Ireland during the pandemic, harm reduction policies were expedited and implemented which meant people in homelessness could access their medication quickly and easily, what policy implications hold for Ontario/Canada? | We thank you for these recommendations and for providing the example from Ireland and Australia (“Discussion”, p 41-42). We further elaborated our recommendations to be more scalable at the HF program level(Table 1, p 47- 48). Moreover, we recommended that strategies that seemed to work better should be maintained beyond pandemic times to increase treatment adherence, completion and health recovery outcomes (Table 1, p 47- 48). |
| Wording: throughout the Discussion the authors use the term “technological inequities” and “social inequities”. I would suggest inequalities instead as equity holds meaning as a legal/financial term and inequality or unfairness is more commonly used in the homelessness literature. | Thank you for the suggestion. We revised the use of these two terms in our “Discussion section”. We feel it is important to highlight that although some of the disparities faced by underserved or socio-economically excluded populations are due to inequalities (imbalance) in financial and social resources, many o such inequalities are caused by inequities or unfairness in the distribution and access to financial, social, material, training, employment, and power resources /opportunities. |
| Typos There is a typo in the title, “undeserved” Pg. 5 line 4, “are is far greater in these…” Pg. 28 “copying” Pg. 32 “staffing” Pg. 35 “clients ‘ “ Error in figure 2. “popultions” | Thank you for these observations. We have proofed the manuscript and addressed the existing typos. |

**Response to the comments from Journal’s editorial team**

We thank the journal’s editorial team for the guideline and suggestions to format the revised manuscript following the journal’s style and structure.

| **Reviewers’ Comments** | **Response** |
| --- | --- |
| Journal requirements:  When submitting your revision, we need you to address these additional requirements.  1. Please ensure that your manuscript meets PLOS ONE's style requirements, including those for file naming. The PLOS ONE style templates can be found at  https://journals.plos.org/plosone/s/file?id=wjVg/PLOSOne_formatting_sample_main_body.pdf and  <https://journals.plos.org/plosone/s/file?id=ba62/PLOSOne_formatting_sample_title_authors_affiliations.pdf>. | Thank you for the journal’s style guideline. We have formatted our revised manuscript to the journal’s style. |
| 2. When reporting the results of qualitative research, we suggest consulting the COREQ guidelines: http://intqhc.oxfordjournals.org/content/19/6/349. In this case, please consider including more information on the number of interviewers, their training and characteristics. | We have made sure our manuscript reporting followed the COREQ guidelines. We enclosed the COREQ Checklist. |
| 3. Thank you for stating the following in the Funding Section of your manuscript:  “The present study received funding from St. Michael’s Hospital Research Training Centre Scholarship awarded (2019) to Dr. Cilia Mejia-Lancheros. The funding institutions had no role in the study design, collection, analysis and interpretation of the data or the preparation, revision, or approval of the present manuscript. The views expressed in this publication are the views of the authors.”  We note that you have provided funding information that is not currently declared in your Funding Statement. However, funding information should not appear in the Acknowledgments section or other areas of your manuscript. We will only publish funding information present in the Funding Statement section of the online submission form.  Please remove any funding-related text from the manuscript and let us know how you would like to update your Funding Statement. Currently, your Funding Statement reads as follows:  “The present study received funding from St. Michael’s Hospital Research Training Centre Scholarship awarded (2019) to Dr. Cilia Mejia-Lancheros. The funding institutions had no role in the study design, collection, analysis and interpretation of the data or the preparation, revision, or approval of the present manuscript. The views expressed in this publication are the views of the authors.”  Please include your amended statements within your cover letter; we will change the online submission form on your behalf. | Thank you for the observation. We have removed the funding statement from the “Acknowledge section” of the revised manuscript. However, we do confirm that we would like that the following statement is reported in the online system:  “The present study received funding from St. Michael’s Hospital Research Training Centre Scholarship awarded (2019) to Dr. Cilia Mejia-Lancheros. The funding institutions had no role in the study design, collection, analysis and interpretation of the data or the preparation, revision, or approval of the present manuscript. The views expressed in this publication are the views of the authors.” |
| 4. Thank you for stating the following in your Competing Interests section:  “The authors declare no competing interests”  Please complete your Competing Interests on the online submission form to state any Competing Interests. If you have no competing interests, please state ""The authors have declared that no competing interests exist."", as detailed online in our guide for authors at http://journals.plos.org/plosone/s/submit-now  This information should be included in your cover letter; we will change the online submission form on your behalf. | Thank you for this observation. We have completed our Competing Interests on the online submission form. As we do not have any competing interests, the competing interest’s statement, now reads as: “The authors have declared that no competing interests exist.” |
| 5. In your Data Availability statement, you have not specified where the minimal data set underlying the results described in your manuscript can be found. PLOS defines a study's minimal data set as the underlying data used to reach the conclusions drawn in the manuscript and any additional data required to replicate the reported study findings in their entirety. All PLOS journals require that the minimal data set be made fully available. For more information about our data policy, please see http://journals.plos.org/plosone/s/data-availability.  Upon re-submitting your revised manuscript, please upload your study’s minimal underlying data set as either Supporting Information files or to a stable, public repository and include the relevant URLs, DOIs, or accession numbers within your revised cover letter. For a list of acceptable repositories, please see http://journals.plos.org/plosone/s/data-availability#loc-recommended-repositories. Any potentially identifying patient information must be fully anonymized.  Important: If there are ethical or legal restrictions to sharing your data publicly, please explain these restrictions in detail. Please see our guidelines for more information on what we consider unacceptable restrictions to publicly sharing data: http://journals.plos.org/plosone/s/data-availability#loc-unacceptable-data-access-restrictions. Note that it is not acceptable for the authors to be the sole named individuals responsible for ensuring data access.  We will update your Data Availability statement to reflect the information you provide in your cover letter.  6. We note that you have indicated that data from this study are available upon request. PLOS only allows data to be available upon request if there are legal or ethical restrictions on sharing data publicly. For more information on unacceptable data access restrictions, please see http://journals.plos.org/plosone/s/data-availability#loc-unacceptable-data-access-restrictions.  In your revised cover letter, please address the following prompts:  a) If there are ethical or legal restrictions on sharing a de-identified data set, please explain them in detail (e.g., data contain potentially sensitive information, data are owned by a third-party organization, etc.) and who has imposed them (e.g., an ethics committee). Please also provide contact information for a data access committee, ethics committee, or other institutional body to which data requests may be sent.  b) If there are no restrictions, please upload the minimal anonymized data set necessary to replicate your study findings as either Supporting Information files or to a stable, public repository and provide us with the relevant URLs, DOIs, or accession numbers. For a list of acceptable repositories, please see http://journals.plos.org/plosone/s/data-availability#loc-recommended-repositories.  We will update your Data Availability statement on your behalf to reflect the information you provide. | Thank you reminded us about your Data availability access policy, which we agreed upon. However, our data could not be made publicly available in any public or accessible repository, as we need to account for privacy, ethical, legal and third-party restrictions. Therefore, we have revised our “Availability of data” and material statement reflecting it. The statement now reads as follows:  “The study data cannot be made publicly available due to the sensitive nature of the data (small study sample with sensitive details, which could lead to participants and their clients’ identification), the agreements and procedures governing using the collected dataset established by the study sponsor, research institution and Research Ethics Board, and the data usage and sharing, and the privacy protection assured to the study’s participants in the consenting process. However, de-identified participant data used in the present study may be made available to investigators who complete the following steps: 1) present a study proposal that has received approval from an independent research committee or research ethics board; 2) provide a data request for review by the study principal investigators (PIs) and co-investigators; 3) following approval of the request, execute a data-sharing agreement between the investigators and the Study PIs. Study proposals and data access requests should be sent to Evie Gogosis (Evie.Gogosis@unityhealth.to), the research manager for the present study, and Dr. Stephen Hwang (Stephen.Hwang@unityhealth.to), principal investigator of the Toronto AH/CS-Quali Study.” |

--------
