# Supplementary material for: Providing Housing First services for an underserved population during the early wave of the COVID-19 pandemic: A qualitative study
Source: PLoS One. 2022 Dec 1;17(12):e0278459. doi: 10.1371/journal.pone.0278459 (PMC9714853; doi:10.1371/journal.pone.0278459)
Supplement: Attachment — Submitted filename: Response to Reviewers_RV2.docx [file pone.0278459.s004.docx]

**Response to Editorial and Reviewers’ Comments**

We thank the editor and reviewers for their comments and suggestions, which improve the quality and readability of our manuscript. We have highlighted changes in yellow in the marked-up copy file.

| **Editorial team’s Comments** | **Response** |
| --- | --- |
| **Journal Requirements:** |  |
| Please review your reference list to ensure that it is complete and correct. If you have cited papers that have been retracted, please include the rationale for doing so in the manuscript text, or remove these references and replace them with relevant current references. Any changes to the reference list should be mentioned in the rebuttal letter that accompanies your revised manuscript. If you need to cite a retracted article, indicate the article’s retracted status in the References list and also include a citation and full reference for the retraction notice | Thank you for requesting the reference list and ensuring the references are all completed and corrected. We have revised the cited references one by one, correcting any format error or updating any publication details (pages 51-56) |
| **Reviewers’ Comments** | **Response** |
| **Reviewer #1:** |  |
| ***Comment #1:***  The authors have sufficiently addressed my feedback and/or provided sufficient justification for their decisions. There remain a number of typos/broken sentences related to the new additions and changes. I would encourage the authors to have a rapid but thorough review to fix these minor concerns. | We thank you for this positive feedback and reassuring comment. As suggested, we have carefully revised and edited the manuscript, addressing existing typos and broken sentences. |
| **Reviewer #2:** |  |
| ***Comment #1:***  It was a pleasure to revisit this manuscript on HF implementation in Toronto during the COVID-19 pandemic. My feelings are the same that the findings reported here are interesting and important for the field of homelessness research, particularly for HF crisis-readiness. I am satisfied that the authors have worked hard to address my comments (and that of other reviewers). I'd just recommend that the authors proofread the manuscript, as I noticed some typos | Thank you for this comment. We agree with the reviewer on the importance and impact our findings could have on homelessness, housing and pandemic crisis readiness and response.  As mentioned above in response to Reviewer #1, we have proofed the manuscript and addressed the remaining typos or editing issues. |
